# Bifunctional spoof surface plasmon polariton meta-coupler using anisotropic transmissive metasurface

**DOI:** 10.1515/nanoph-2021-0761

**Published:** 2022-02-15

**Authors:** Dengpan Wang, Kaiyue Liu, Xiaofeng Li, Guangming Wang, Shiwei Tang, Tong Cai

**Affiliations:** Air and Missile Defense College, Air Force Engineering University, Xi’an 710051, China; Information Engineering University, Zhengzhou, 450001, China; School of Physical Science and Technology, Ningbo University, Ningbo 315211, China; State Key Laboratory of Modern Optical Instrumentation, The Electromagnetics Academy Zhejiang University, Hangzhou 310027, China

**Keywords:** bifunctional meta-coupler, spoof surface plasmon polaritons, transmissive metasurface, wavefront manipulation

## Abstract

Tailoring the wavefronts of spoof surface plasmon polaritons (SSPPs) at will, especially with multifunctional integration, is of great importance in near-field photonics. However, conventional SSPP devices suffer from the issues of bulk configurations, limited functionalities, and single operating modes, which are unfavorable for electromagnetic (EM) integration. Here, a novel scheme is proposed to design bifunctional SSPP meta-devices based on the polarization dependent property via satisfying the comprehensive phase distributions and multi-mode momentum matching in a transmission geometry. As proof of the concept, we experimentally demonstrate a bifunctional SSPP meta-device in the microwave regime that can convert incident *x*- and *y*-polarized waves to transverse magnetic (TM)-mode SSPP Bessel beams and transverse electric (TE)-mode SSPP focusing beams, respectively. Our findings open a door to achieve near-field manipulation of SSPPs with multi-function and multi-mode integration, which can stimulate the applications of SSPP functional devices, such as near-field sensing, imaging, and on-chip photonics.

## 1 Introduction

Spoof surface plasmon polaritons (SSPPs), the low-frequency (e.g., THz and GHz) counterparts of natural surface plasmon polaritons (SPPs), are the electromagnetic (EM) eigenmodes that bounded at dielectric/metal interfaces, which exhibit the extraordinary properties of sub-wavelength resolution and local field enhancement [1], [2], [3], [4], [5], [6], [7], [8]. SSPPs have displayed many fascinating applications, such as in antennas [9], [10], [11], [12], integrated wireless communication systems [13], waveguides [14], imaging [15, 16], sub-wavelength circuits [17], and near-field photonics areas [18]. Generally, to achieve those promising applications, it is necessary to excite the SSPPs with tailored wavefronts efficiently and flexibly. Traditionally, prisms or gratings are employed to excite the SSPPs, while transportation phase accumulations or Bragg scatterings with grooves and slits patterned on SSPP eigenmode plates are utilized to tailor the wavefronts of SSPPs [19], [20], [21], [22], [23]. However, these methods have the disadvantages of bulk configurations, limited functionalities, and single operating modes, which are unfavorable for highly integrated modern devices [24].

Recently, multifunctional metasurfaces have attracted a great deal of attention due to their powerful wavefront manipulation capability, yielding various fascinating propagation wave (PW) manipulation effects, such as full-space manipulation [24], [25], [26], spin-decoupling or selection [27], [28], [29], [30], information- or time-space coding [31], [32], [33], [34], and many others [35], [36], [37], [38], [39], [40], [41], [42], [43], [44], [45], [46], [47]. Meanwhile, high-efficiency SSPP meta-couplers based on metasurfaces have been proposed, including unidirectional SSPP meta-couplers [48], [49], [50], [51], [52] and polarization-controlled tunable and functional meta-couplers [53], [54], [55], [56], [57], but the impossibility of SSPP wavefront manipulation limits their applications. The trend in modern science and technology has been to implement SSPP multifunctional integration with arbitrary wavefronts and modes. In 2018, plasmon metal plates, together with an additional reflective all-dielectric metasurface, were employed to manipulate the wavefront of the excited SSPP [58]. Very recently, a kind of ultra-thin reflective metasurface with Pancharatnam–Berry (PB) phase efficiently excited transverse magnetic (TM)-mode SSPPs with tailored wavefronts [59, 60]. Despite these achievements, most of the reported meta-devices are limited by their single SSPP mode (only the TM mode), since it is difficult to support a transverse electric (TE) SSPP mode for a negative permeability medium with the momentum-matching requirement of the SSPP eigenmode plate. Meanwhile, it is promising to excite and match the momentum of a multi-mode and multi-function SSPP in a transmission geometry, because the obtained field avoids interference with the incident waves.

**Figure 1:**
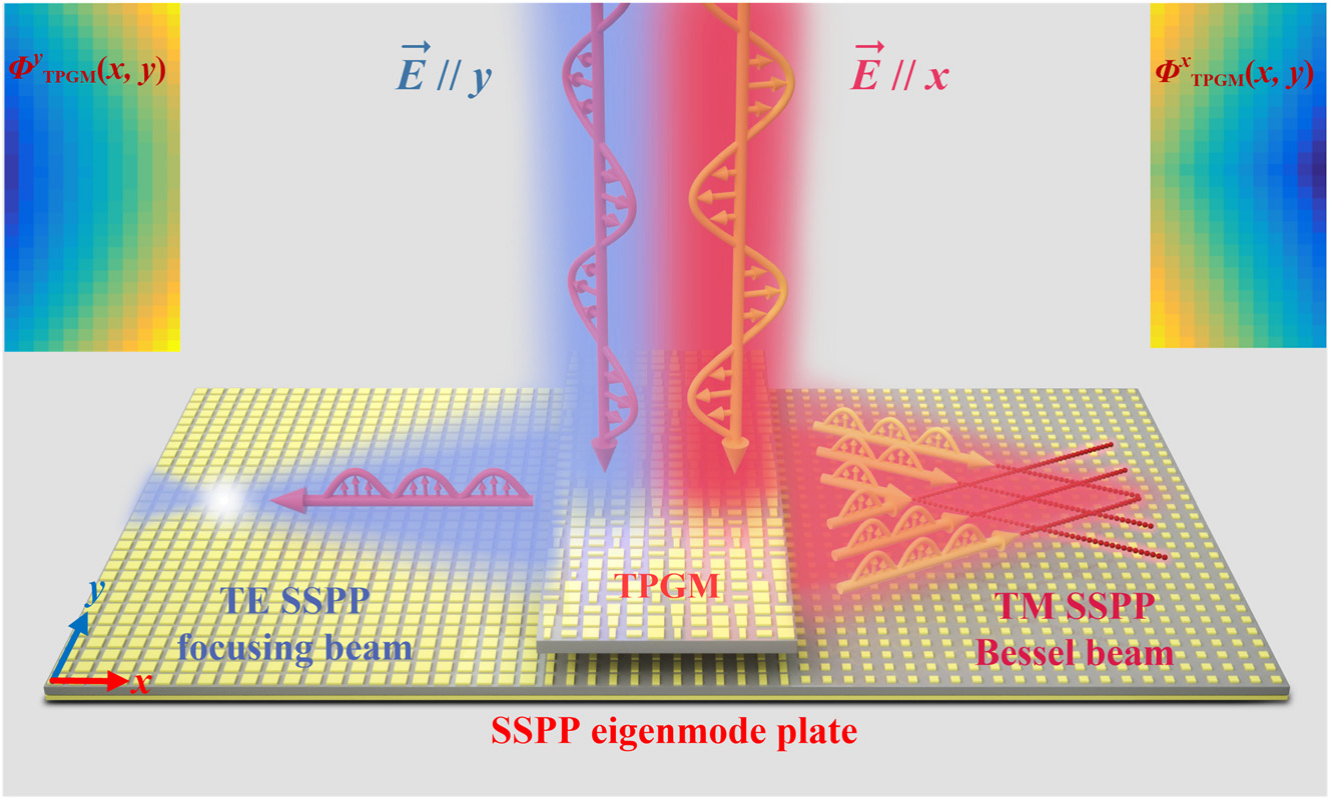
Schematic of the designed bifunctional SSPP meta-coupler. When the *y*-polarized plane wave is incident on the top layer (TPGM), the TE-mode SSPP focusing beam on the bottom layer is generated, which travels to the left. For the *x*-polarized incident plane wave, the TM-mode SSPP Bessel beam is excited and flows to the right side. The two inset color maps are the theoretical phase distributions of *y* polarization and *x* polarization for the TPGM.

In this paper, we propose a novel scheme to efficiently excite both TM- and TE-mode SSPPs with arbitrary wavefronts integrated on a single device in the transmission geometry. For verification, a bifunctional SSPP meta-coupler is designed, fabricated, and experimentally demonstrated in the microwave regime. This coupler efficiently converts incident *x*- and *y*-polarized waves to TM-mode SSPP Bessel beams and TE-mode SSPP focusing beams with opposite directions of propagation, as schematically illustrated in Figure 1. The anisotropic transmissive metasurfaces are employed to satisfy the desired multifunctional SSPP phase distributions with high transmission amplitudes and the dispersion-controlled SSPP eigenmode plate is introduced to match the momentum of the multi-mode SSPP. Our findings substantially enrich the application scenarios of SSPP near-field manipulation with multi-function and multi-mode integration.

## 2 Principle and design

### 2.1 Working principle of the bifunctional SSPP meta-coupler

To excite SSPPs with high efficiency, scientists usually designed metasurfaces with a linear phase profile $\Phi \left(x,y\right)=\varphi_{0}+\xi_{x}x$ [2, 3], with $\xi_{x}$ being the phase gradient along *x*-direction satisfying $\xi_{x}>k_{0}$ , and $\varphi_{0}$ being the initial phase with $\varphi_{0}=\text{constant}$ (see Figure S1(a) in Supplementary Material). The excited SSPP above can only propagate along the phase gradient direction without generating further EM functions. Therefore, to excite the SSPP and simultaneously control its wavefront arbitrarily, the initial value $\varphi_{0}$ should be regarded as a phase function $\varphi_{0}\left(y\right)$ (see details in Figure S1(b) in Supplementary Material). The various fascinating near-field wave-manipulation phenomena of SSPPs (e.g., SSPP beam deflection, beam focusing, and Bessel beams) can be achieved by configuring the phase function $\varphi_{0}\left(y\right)$ . Take an SSPP focusing beam as an example. Here, $\varphi_{0}\left(y\right)=k_{\text{sspp}}\left(\sqrt{y^{2}+F^{2}}-F\right)$ should be satisfied, in analogy with the focusing phase distribution of PWs. Then, the phase profile for SSPP can be described as:

(1) $$\Phi \left(x,y\right)=\xi_{x}x+k_{\text{sspp}}\left(\sqrt{y^{2}+F^{2}}-F\right)$$

where $\xi_{x}=k_{\text{sspp}}$ to match the wave vector of the SSPP eigenmode plate. When illuminated by a normally incident EM wave, an SSPP focusing beam can be generated with the focal point at a distance of *F*.

To implement TM- and TE-mode SSPPs with two different functions on a single meta-device as shown schematically in Figure 1, the whole device is composed of two layers of plates. The top layer is composed of an anisotropic transmissive phase gradient metasurface (TPGM) with different phase profiles for *x*- and *y*-polarized waves. The comprehensive phase distribution of this anisotropic TPGM can be expressed as:

(2) $$\left\{ \begin{array}{c} \Phi_{\text{TPGM}}^{x}\left(x,y\right)=\xi_{x}x+\varphi_{0}^{x}\left(y\right)\\ \Phi_{\text{TPGM}}^{y}\left(x,y\right)=-\xi_{x}x+\varphi_{0}^{y}\left(y\right) \end{array}\right.$$

where $\Phi_{\text{TPGM}}^{x}\left(x,y\right)$ and $\Phi_{\text{TPGM}}^{y}\left(x,y\right)$ are the phase distributions of the TPGM for both polarizations, and $\varphi_{0}^{x}\left(y\right)$ and $\varphi_{0}^{y}\left(y\right)$ are the corresponding functional phases. Therefore, to create TM- and TE-mode SSPP bifunctional meta-device with flexibly tailored wavefronts in the transmission geometry, it is necessary to design a metasurface possessing arbitrary desired phase responses under the excitation of both *x*- and *y*-polarized waves.

As we all know, gradient phase metasurfaces are a good choice for SSPP couplers, but they cannot guide to form the SSPPs because the energy is bounded on the metasurfaces. As a result, we need to design an SSPP eigenmode plate to form and support the propagation of the SSPPs. For the reflective SSPP coupler, the SSPP eigenmode plates are always placed next to the SSPP coupler made from a gradient phase metasurface [2]. However, for such a setup, the TE mode SSPP is difficult to couple out to the SSPP eigenmode plate, even if the SSPP eigenmode plate supports that mode. To solve this problem, we designed a transmissive SSPP coupler in which the SSPP eigenmode plate is placed on the bottom layer, which can couple out both the TM and TE modes of SSPPs and also support their propagation.

### 2.2 Transmissive metasurface and SSPP eigenmode plate designs

**Figure 2:**
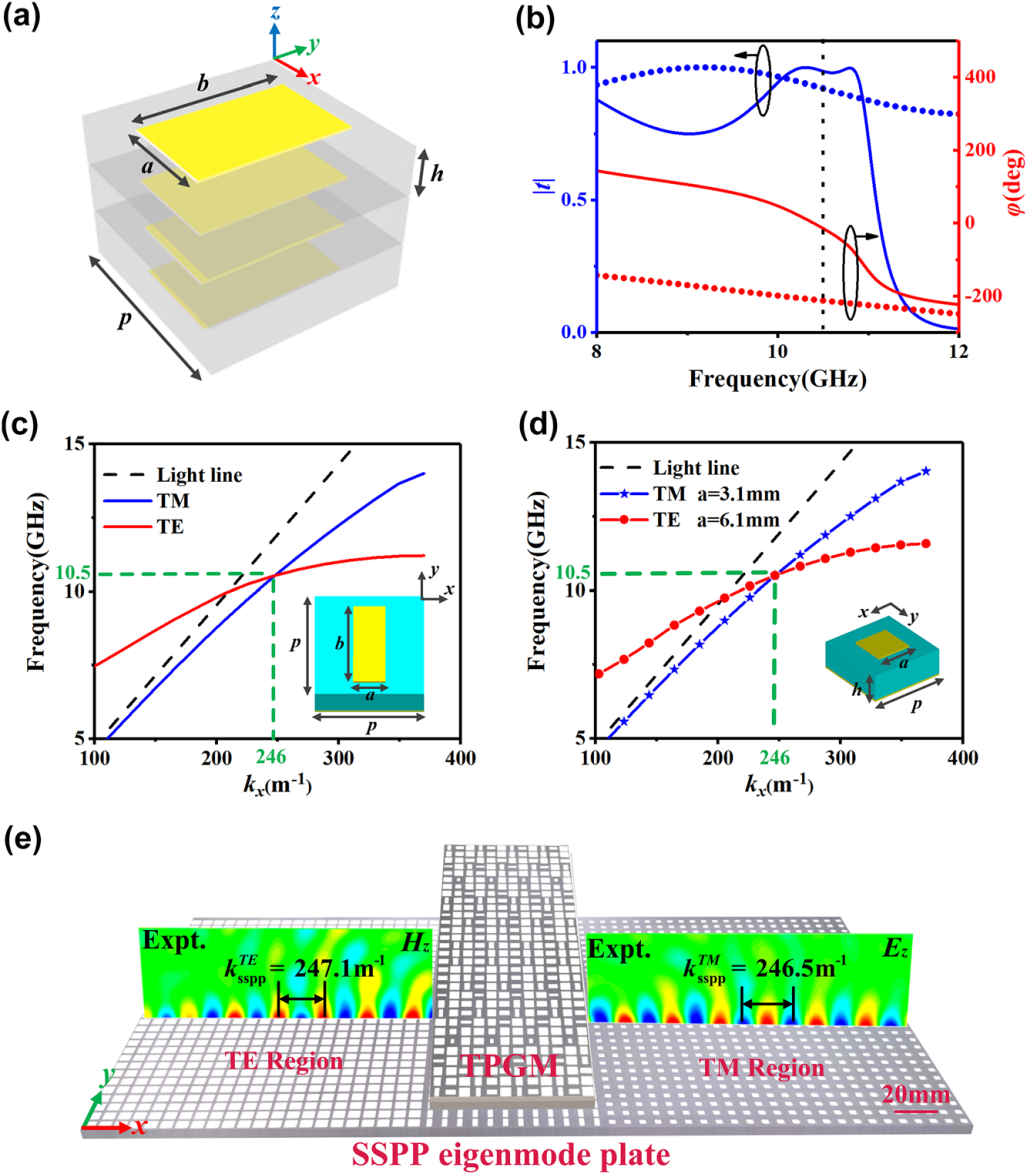
The design and performance of transmissive metasurface and SSPP eigenmode plate. (a) Schematic of the composite metasurface. The substrate is composed of F4B with relative permittivity 3.5, loss tangent 0.002, period *p* = 8.5 mm, and thickness *h* = 2 mm. The metal is copper with conductivity *σ* = 5.8 × 10^7^ S m^−1^ and thickness *t* = 0.036 mm. The parameters *a* and *b* are tuned based on the phase distributions. (b) The transmission amplitude and phase spectra of the metasurface (*a* = 4.1 mm, *b* = 6.4 mm) under excitation of *x*-polarized (dotted line) and *y*-polarized (solid line) waves, as obtained by FDTD simulations. (c) and (d) The FDTD simulation dispersion relation of (c) one anisotropic eigen-unit and (d) two isotropic eigen-units with the structures depicted in the inset. The anisotropic eigen-unit consists of a metallic patch on the top with parameters *a* = 2.5 mm and *b* = 6.5 mm, while the two isotropic eigen-units consist of two different square metallic patches on the top with parameters *a* = 3.1 mm (TM-mode isotropic eigen-unit) and *a* = 6.1 mm (TE-mode isotropic eigen-unit), respectively. Here, all the eigen-units are composed of metallic patches with different structural parameters and a metallic ground plane separated by a 3 mm thick dielectric substrate (*ε*_*r*_ = 2.65 + 0.002i, with periodicity *p* = 8.5 mm). (e) Experimentally measured Re(*H*_*z*_) and Re(*E*_*z*_) field distributions on part of the fabricated SSPP eigenmode plate on the *x*–*z* plane when the meta-device is, respectively, illuminated by *y*-polarized and *x*-polarized incident waves at 10.5 GHz.

Figure 2(a) shows the proposed composite metasurface composing of four metallic layers separated by three dielectric substrates. The metasurface exhibits global mirror symmetry, and its EM characteristics can thus be described by two diagonal Jones matrices $R=\left(\begin{array}{cc} r_{xx} & 0\\ 0 & r_{yy} \end{array}\right)$ and $T=\left(\begin{array}{cc} t_{xx} & 0\\ 0 & t_{yy} \end{array}\right)$ , where $r_{xx}\left(r_{yy}\right)$ and $t_{xx}\left(t_{yy}\right)$ are, respectively, the reflection and transmission coefficients for *x*-polarized (*y*-polarized) waves. According to the multi-resonant Lorentz model in Refs [3, 24, 61], a multi-layer system of stacked resonant metal patches significantly increases the transparency window and transmission-phase variation range. Figure 2(b) presents metasurface’s transmission amplitude and phase spectra (i.e., $\left|t_{xx}\right|$ , $\left|t_{yy}\right|$ , $\left|\varphi_{xx}\right|$ , and $\left|\varphi_{yy}\right|$ ), which shows that the transmission amplitudes exceed 0.8 within 8–11 GHz and the phase variation range covers 360°. To further provide the comprehensive phase profiles Φ(x,y) of our bifunctional SSPP meta-device, the two-dimensional amplitude and phase distributions at a working frequency of 10.5 GHz were simulated by the FDTD method (see Section B, Supplementary Material). Based on the two-dimensional amplitude and phase distributions obtained above, we can design the anisotropic TPGM to provide the required comprehensive phase distributions $\Phi_{x}\left(x,y\right)$ and $\Phi_{y}\left(x,y\right)$ at the same time for our bifunctional SSPP meta-device.

Traditionally, to support both TM- and TE-mode SSPPs coupled out from the phase gradient metasurface, an anisotropic SSPP eigenmode plate is necessary to match the momentum of the SSPP. With the wave-vector $k_{\text{sspp}}=1.12k_{0}$ at 10.5 GHz to match the phase gradient of the TPGM, the dispersion relation of the anisotropic unit is plotted in Figure 2(c), which shows that the metasurface supports both TM- and TE-mode SSPPs at 10.5 GHz coupled from the TPGM of the upper layer.

Such an anisotropic SSPP eigenmode plate is sufficient for the conventional planar wavefront SSPP. However, because of the momentum mismatch, the anisotropic SSPP eigenmode plate does not support the oblique propagation of the SSPP very well when we try to manipulate the wavefront of the SSPP (see Section C, Supplementary Material). To resolve this problem, we design two kinds of SSPP eigenmode plates made of isotropic eigen-units to match the TM- and TE-mode SSPPs, respectively. The dispersion relations of the isotropic eigen-units are plotted in Figure 2(d), and the simulations show that all the eigen-units can support the theoretically excited SSPP at 10.5 GHz. As the fabricated sample in Figure 2(e) illustrates, two isotropic SSPP eigenmode plates are placed on each side of the anisotropic SSPP eigenmode plate to receive the SSPPs with special wavefronts.

Moreover, Figure 2(e) illustrates the experimentally measured $\text{Re}\left(H_{z}\right)$ for the TE-mode SSPP and $\text{Re}\left(E_{z}\right)$ for the TM-mode SSPP on part of the fabricated isotropic SSPP eigenmode plate on the *x*–*z* plane (with *y* = 0) when the meta-device is, respectively, illuminated by *y*-polarized and *x*-polarized incident waves at 10.5 GHz. The figure demonstrates that the field distributions represent a very well-defined SSPP with obvious local field enhancement. Meanwhile, the measured wave vectors of $\text{Re}\left(H_{z}\right)$ and $\text{Re}\left(E_{z}\right)$ at 10.5 GHz are, respectively, $k_{\text{sspp}}^{\text{TE}}=247.1\;{\text{m}}^{-1}$ and $k_{\text{sspp}}^{\text{TM}}=246.5\;{\text{m}}^{-1}\text{,}$ which agrees well with the theoretically predefined value $k_{\text{sspp}}=246.3\;{\text{m}}^{-1}$ .

## 3 Meta-device implementation

### 3.1 Meta-device design

With the transmissive metasurface and SSPP eigenmode plate in hand, we next design a dual-mode SSPP bifunctional meta-coupler integrating the Bessel beam and focusing beam effects. The Bessel beam is a special type of nondiffraction beam with unique self-healing properties, attracting much interest in recent years. This beam exhibits great potential applications in near-field probing, manipulation, and so on [62]. As for the focusing beam, it can converge EM waves to one point to achieve high directivity, and is widely used in antennas and other fields [26].

**Figure 3:**
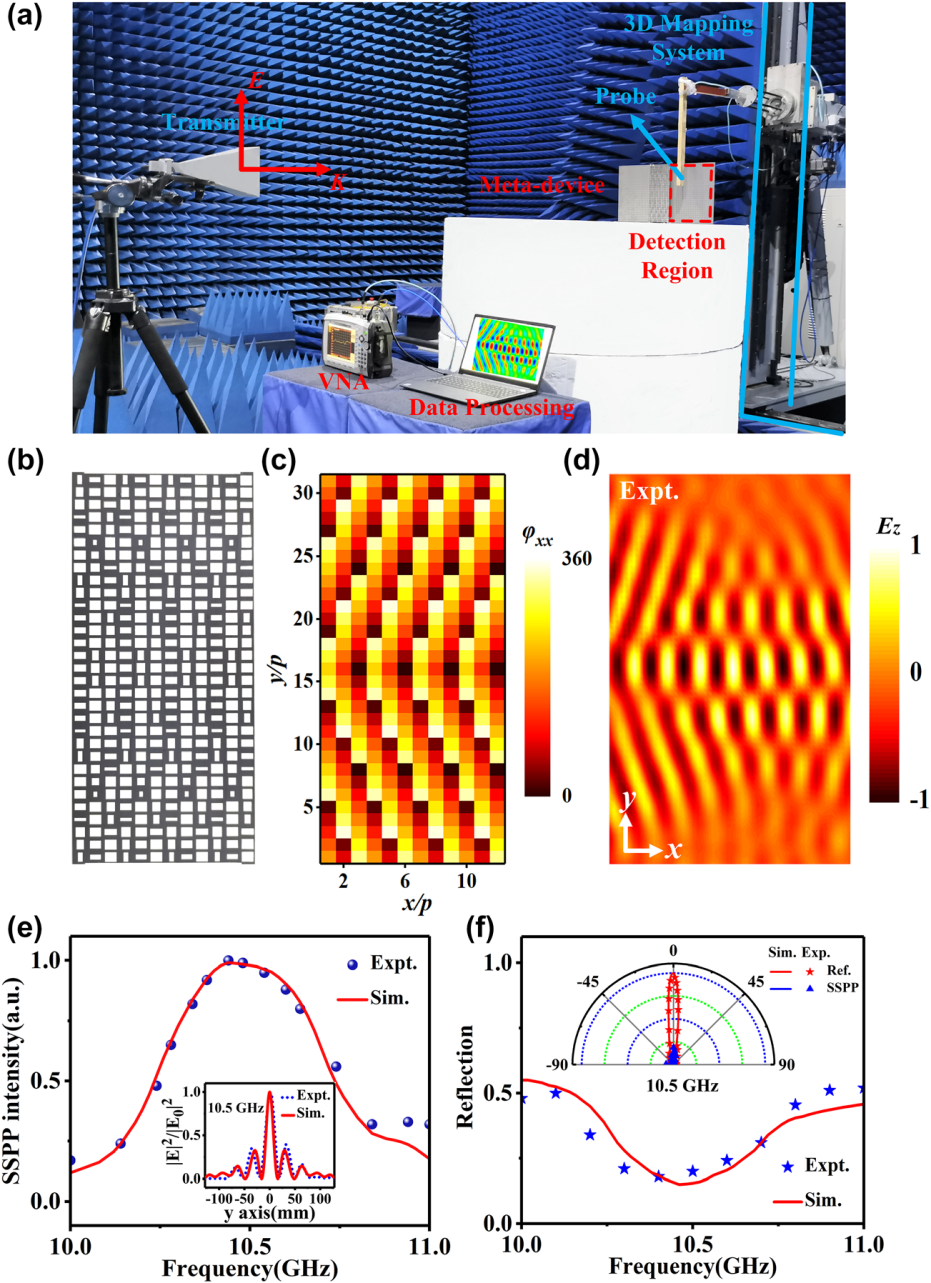
Design and experimental demonstration of the proposed meta-device with SSPP Bessel beam. (a) Near-field experimental setup and schematic diagram of the fabricated prototype meta-device with an anisotropic TPGM and a composite SSPP eigenmode plate. (b) The fabricated prototype of the TPGM. (c) Theoretically calculated and FDTD-simulated phase profiles of $\varphi_{xx}$ encoded in the proposed metasurface. (d) Experimentally measured $\text{Re}\left(E_{z}\right)$ field distributions on a plane 6 mm above the right side of the SSPP eigenmode plate. (e) Simulated and measured SSPP intensity of the Bessel beam. The inset is the field distribution of the Bessel beam versus the *y* coordinate at 10.5 GHz. (f) Simulated and measured total reflection of the SSPP Bessel beam with the scattered field curves of the meta-device and the comparison metallic plate at 10.5 GHz depicted in the inset.

**Figure 4:**
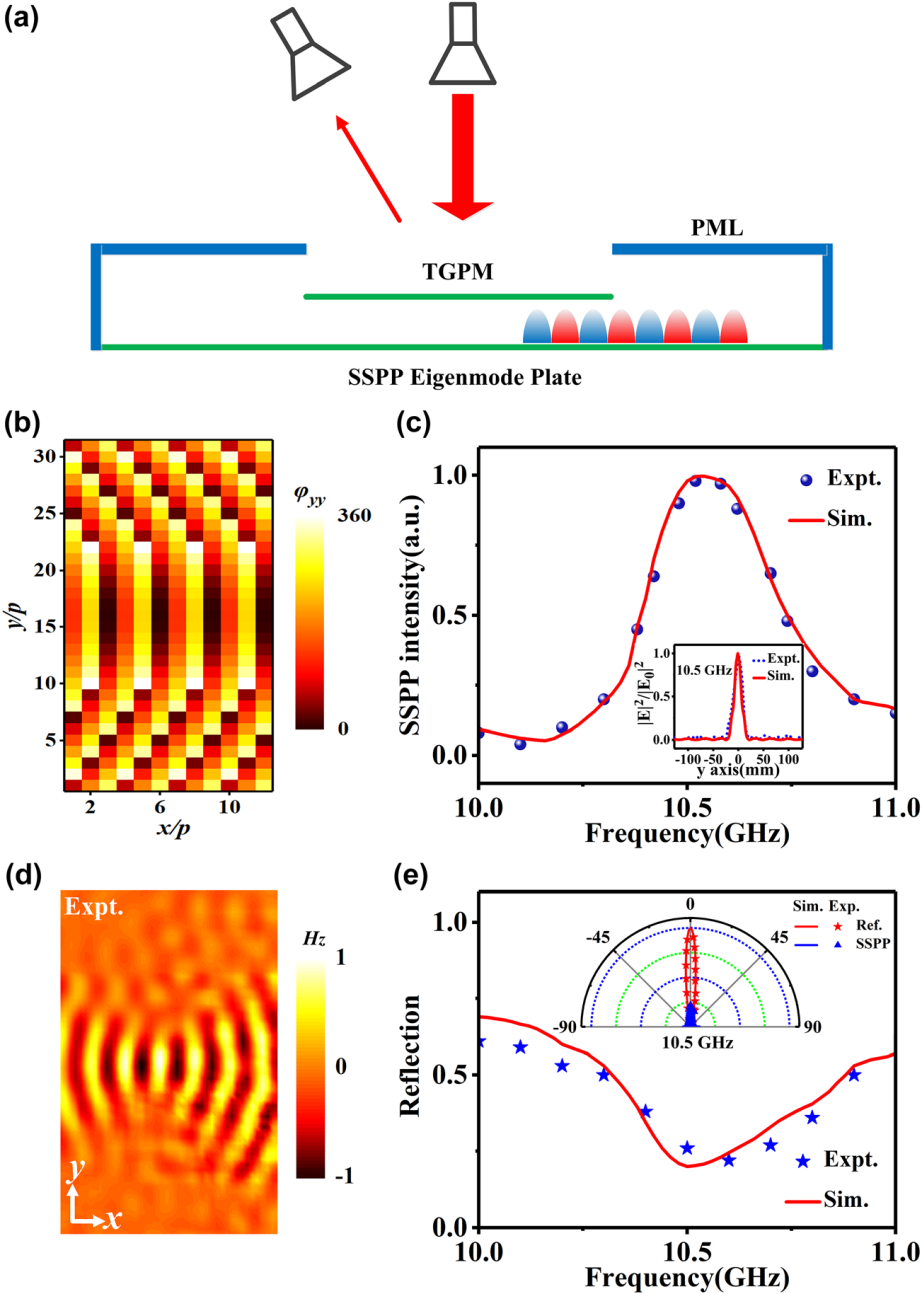
Design and experimental demonstration of the proposed meta-device with SSPP focusing beam. (a) Schematic of the far-field experimental setup. (b) Theoretical and FDTD-designed phase profiles on the TPGM. (c) Simulated and measured SSPP intensity of the SSPP focusing beam versus frequency. The inset shows the field distribution at the focal length position versus the *y* coordinate with *F* = 140 mm at 10.5 GHz. (d) Experimentally measured $\text{Re}\left(H_{z}\right)$ field distributions on the *x*–*y* plane at the frequency of 10.5 GHz, as excited by a *y*-polarized wave. (e) Simulated and measured total reflection versus the frequency for the TE-mode SSPP focusing beam. The inset shows the scattered field curve of the meta-device and the comparison metallic plate at 10.5 GHz.

To achieve bifunctional integration, the anisotropic TPGM exhibits the following comprehensive phase distributions at 10.5 GHz:

(3) $$\left\{ \begin{array}{c} \Phi_{\text{TPGM}}^{x}\left(x,y\right)=\xi_{x}x+\xi_{y}\left|y\right|\\ \Phi_{\text{TPGM}}^{y}\left(x,y\right)=-\xi_{x}x+k_{\text{sspp}}\left(\sqrt{y^{2}+F^{2}}-F\right) \end{array}\right.$$

where $\xi_{x}=k_{\text{sspp}}=1.12k_{0}$ , $\xi_{y}=0.42k_{0}$ , and *F* = 150 mm. Using Eq. (3), the theoretical phase distributions for $\Phi_{\text{TPGM}}^{x}\left(x,y\right)$ and $\Phi_{\text{TPGM}}^{y}\left(x,y\right)$ at each point occupied by a metasurface on the TPGM are shown in Figures 3(c) and 4(b). We can unambiguously determine the dimensions *a* and *b* of all units based on the two-dimensional amplitude and phase distributions (Section B, Supplementary Material). The distributions of parameters *a* and *b* of these metasurfaces are depicted in Section B, Supplementary Material. Figure 3(b) depicts the fabricated TPGM, which contains 12 × 31 meta-atoms with a total area of 102 × 263.5 mm. The fabricated sample illustrates that each column of the TPGM consists of a meta-atom array that yields the phase gradient $\pm \xi_{x}$ ; meanwhile, the initial meta-atoms of different columns are of different initial phases generating the functions $\varphi_{0}\left(y\right)$ , which collectively yield the desired SSPP Bessel (focusing) beam phase profiles under the excitation of *x*-polarized (*y*-polarized) waves. Next, we placed the designed TPGM just above the composite SSPP eigenmode plate, which occupies a total size of 493 × 263.5 mm.

### 3.2 Experimental verification

We fabricated a prototype meta-device containing an anisotropic TPGM and a composite SSPP eigenmode plate, and experimentally investigated its near- and far-field performance in a microwave anechoic chamber, as depicted in Figure 3(a).

We first experimentally characterized the performance of the SSPP Bessel beam. The TPGM was illuminated normally by an *x*-polarized wave emitted from a horn antenna, and a monopole antenna was employed to detect the local $\text{Re}\left(E_{z}\right)$ field patterns for the TM-mode SSPP on the *x*–*y* plane (with *z* = 6 mm) at the right side of the meta-device, as illustrated in Figure 3(a). Both monopole antenna and horn antenna were connected to a vector network analyzer (Agilent E8362C PNA), linked with a computer for data processing. Figure 3(d) plots the experimentally measured $\text{Re}\left(E_{z}\right)$ field distributions obtained on the *x*–*y* plane at frequency 10.5 GHz. The figure shows that most incident *x*-polarized waves generate the SSPP Bessel beam on the right side of the meta-device. The Bessel beam is formed by the interference of two oppositely deflected SSPP beams, which is in good agreement with the theoretical design and simulation (see Section D, Supplementary Material). The simulated crosstalk fields of the SSPP Bessel beam are also depicted in Section D of the Supplementary Material, which are very weak compared with the main polarization results since our composite SSPP eigenmode plate has isotropic eigen-units on both sides for TM SSPP Bessel and TE SSPP focusing beam, respectively. Moreover, the SSPP intensity was calculated through the $E_{z}$ distributions obtained above, as depicted in Figure 3(e). The SSPP intensity curve illustrates that its maximum value appears at 10.5 GHz, which verifies the design correctness and good performance of the SSPP meta-device. More importantly, to further characterize the performance of our SSPP Bessel beam, the inset to Figure 3(e) shows the field intensity distributions of the beam at the *y*-axis, which exhibits the exact characteristics of the Bessel function distribution. Therefore, we have demonstrated that the incident *x*-polarized wave is efficiently converted into an SSPP Bessel beam, agreeing well with the theoretical results.

Then, we quantitatively characterized the performance of our bifunctional meta-device through far-field experiments. As schematically illustrated in Figure 4(a), the incident wave goes to three channels: conversion into SSPP, scattering to the far-field (i.e., total reflection *R*), and structure absorption. Here, the structure absorption is mainly determined by the loss of the dielectric layers, and an approximate estimation value can be obtained in the simulations [3]. Therefore, the SSPP conversion efficiency, an important parameter that quantitatively describes the incident energy converted into SSPP, is mainly determined by the scattered energies. To quantitatively describe the scattered energy (i.e., total reflection), we first illuminated the meta-device by an *x*-polarized wave emitted from a horn antenna (8–12 GHz) and then used another horn antenna to detect the radiation patterns at different angles ranging from −90° to 90°. The inset in Figure 3(f) shows the normalized (to the metal) far-field scattering distributions of the meta-device at 10.5 GHz. As a comparison, the normalized (to the maximum value) scattering of a metallic plate with the same size as the excitation region is also given in the inset. The measured results, coinciding well with the simulated ones, show that the radiation pattern of the meta-device is drastically suppressed compared with that of the metallic plate. Moreover, we integrate the scattered waves over all angles at different frequencies to obtain the total reflected power at the corresponding frequencies, while the total incident power is calculated by integrating over the scattered energy with the excitation area covered by an equal-sized metallic plate. The ratio of the two powers describes the total reflection *R* of the meta-device quantitatively, as shown in Figure 3(f). Both the measured and simulated total reflection (*R*) curves have an obvious trough with *R* only about 0.17 at 10.5 GHz, indicating that the total reflection of the meta-device is very weak and the incident energy is mainly converted into SSPP. Furthermore, we have investigated the total working efficiency of the TM SSPP Bessel beam with the maximum value of 0.6, which is defined as the ratio between the power carried by the SSPP functional beam and that carried by the incident power (see Section E, Supplementary Material). Meanwhile, the crosstalk efficiency of the TM SSPP Bessel beam has also been investigated with a value of 2% at 10.5 GHz, which illustrates that there is little energy will be lost in another channel (see Section B, Supplementary Material).

Next, the focusing effect of the meta-device was investigated, using an experimental setup that is quite similar to that of the Bessel beam. Illuminating the TPGM by a normally *y*-polarized wave, we then utilized a coil magnetic antenna to detect the local $\text{Re}\left(H_{z}\right)$ field patterns for the TE-mode SSPP at the left side of the same plate, as depicted in Figure 4(d). The excited SSPP converges to the pre-designed focal point, in good agreement with the simulations (see Section D, Supplementary Material with both simulations and crosstalk results). Moreover, Figure 4(c) and (e) show the performance of our SSPP focus beam and the far-field results of the meta-device, which exhibits similar conclusions as drawn for the aforementioned TM-mode results. At the frequency of 10.5 GHz, the SSPP intensity detected on the SSPP eigenmode plate also reaches its maximum. At the same time, the scattering mode of the SSPP focusing beam is also suppressed, and the total reflection *R* of the measurement and simulation are only roughly 0.2. Meanwhile, the inset in Figure 4(c) shows the field intensity distribution of the focused SSPP at the focal line, which exhibits the excellent performance of our focusing beam. Both the measured and simulated results in the inset illustrate that the focal spot size (defined as half-power beamwidth) is equal to 14 mm ( $0.49\lambda_{0}$ at 10.5 GHz) and the maximum value appears in the middle of the *y*-axis, which indicates that the energy converted into the SSPP is then converged into the focal point. The working and crosstalk efficiency of the TE SSPP focus beam at 10.5 GHz is 57 and 4%, which is quantitatively described by the SSPP focused working and crosstalk energy (see Section E, Supplementary Material). Finally, we note that the distance *d* between the TPGM and the SSPP eigenmode plate has an important influence on the SSPP efficiency, and the highest efficiency can be obtained when *d* is fixed at a particular value *d*_c_ = 12 mm. (see Section F, Supplementary Material).

## 4 Conclusions

In this paper, a novel meta-coupler is presented, which achieves multi-function and multi-mode integration in the near-field manipulation of SSPP in a transmission system. Following the new design scheme, the proposed meta-device can, respectively, convert incident *x*- and *y*-polarized waves to TM-mode SSPP Bessel beams and TE-mode SSPP focusing beams with opposite propagating directions at 10.5 GHz. Moreover, due to the flexible designs of the phase distributions of the TPGM, arbitrary functions can be integrated on a single meta-device in the transmission system, e.g., SSPP deflected beam, focusing beam, or Bessel beam. With arbitrary functions and modes as well as multifunctional integration, the proposed single meta-device has great potential in near-field photonics working at other frequency spectra.

## Supplementary Material

Supplementary Material
