# Supplementary material for: Bifunctional spoof surface plasmon polariton meta-coupler using anisotropic transmissive metasurface
Source: Nanophotonics. 2022 Feb 15;11(6):1177–85. doi: 10.1515/nanoph-2021-0761 (PMC11501855; doi:10.1515/nanoph-2021-0761)
Supplement: Supplementary file 1 — Supplementary Material [file j_nanoph-2021-0761_suppl.docx]

**Supplementary Material**

**Bifunctional spoof surface plasmon polariton meta-coupler using anisotropic transmissive metasurface**

Dengpan Wang^1, 5^, Kaiyue Liu^3, 5^, Xiaofeng Li^1^, Guangming Wang^1, *^, Shiwei Tang^4, *^ and Tong Cai^1, 2, *^

*^1^Air and Missile Defense College, Air Force Engineering University, Xi’an, 710051, China*

*^2^State Key Laboratory of Modern Optical Instrumentation, the Electromagnetics Academy Zhejiang University, Hangzhou, 310027, China*

*^3^Information Engineering University, Zhengzhou, Henan 450001, China*

*^4^School of Physical Science and Technology, Ningbo University, Ningbo 315211, China*

*^5^These authors contributed equally*

**Corresponding Authors: caitong326@sina.cn (Tong Cai), tangshiwei@nbu.edu.cn (Shiwei Tang), wgming01@sina.com (Guangming Wang)*

A. Principle of phase control for the SSPP meta-couplers............................................2

B. Design and detailed structural parameters of the TPGM..........................................2

C. Design and details of the composite SSPP eigenmode plate....................................4

D. Simulation results and cross talk for the bifunctional SSPP meta-coupler..............5

E. The efficiency of the bifunctional SSPP meta-coupler.............................................7

F. Simulated efficiency of the meta-coupler versus the distance *d* between the metasurface and the SSPP eigenmode plate..................................................................9

1. **Principle of phase control for the SSPP meta-couplers**

As we all know, SSPP is eigen EM modes bounded at dielectric/metal interfaces with wave vector $k_{\mathrm{sspp}}$ larger than the free-space value $k_{0}$. As discussed in the main text, here we design a metasurface with a linear phase profile $\Phi(x, y)=\varphi_{0}+\xi_{x}x$ to excite the SSPP with high efficiency, as illustrated in Fig. S1(a). The phase gradient $\xi_{x}$ provided by TPGM is larger than $k_{0}$ and an SSPP eigenmode plate is designed to guide the SSPP. Moreover, we further manipulate the $\varphi_{0}$ in Fig. S1(a) as a functional phase function $\varphi_{0}(y)$ to realize arbitrary functional SSPP meta-coupler. Figure S1(b) depicts a focusing SSPP meta-coupler with $\varphi_{0}(y)$ as a focusing phase function.


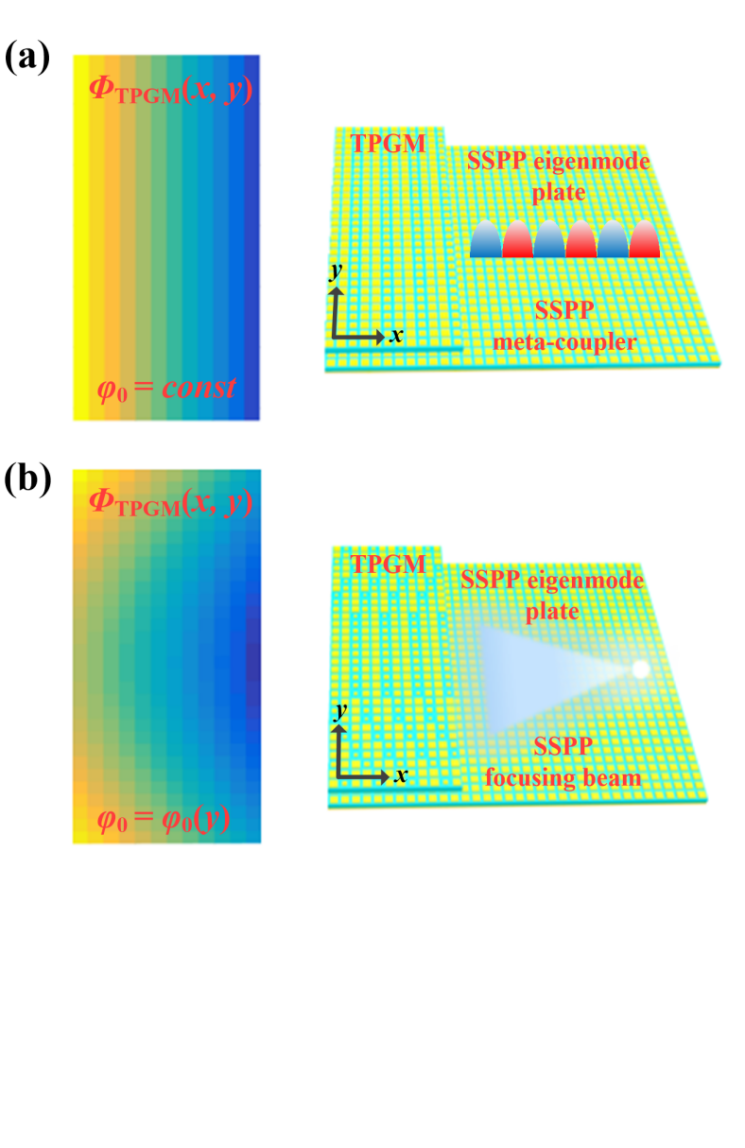


**Figure S1 The principle of phase control for different SSPP meta-couplers.** The phase distributions of TPGM for (a) SSPP meta-coupler with $\varphi_{0}=constant$, (b) functional SSPP meta-coupler with $\varphi_{0}=\varphi_{0}(y)$. Here, we just take an SSPP focusing beam as an example.

1. **Design and detailed structural parameters of the TPGM**

As discussed in the main text, to design the bifunctional SSPP meta-coupler, it should be designed that the 2D TPGM with two different transmission-phase distributions for *x* and *y*-polarized incidence waves respectively. The TPGM is designed as the following two steps. First, the relationships between the transmission coefficients and geometrical parameters *a* and *b* at working frequency are analyzed and calculated. Figure S2(a)-(d) depict the two-dimensional amplitude and phase distributions (i.e., $|t_{xx}|$, $|t_{yy}|$, $\left| \varphi_{xx} \right|$ and $|\varphi_{yy}|$) of the metasurface at 10.5 GHz by FDTD simulations. It can be seen that $\varphi_{xx}(\varphi_{yy})$ is quite sensitive to parameter *a* (*b*) but is quite insensitive to parameter *b* (*a*) and the phase variation covers 360°*.* More importantly, $|t_{xx}|$ and $|t_{yy}|$ all maintain high transmittance (both exceed 0.8) with *a* and *b* variation. Then, according to the needed composite transmission-phase distributions in Figs. 3(a) and 3(c) and aided by Figs. S2(a)-(d), we can obtain the values of *a* and *b* for all metasurfaces to design the TPGM. The distributions of parameters $a(x,y)$ and $b(x,y)$ of the TPGM are illustrated in Figs. S2(e) and S2(f).


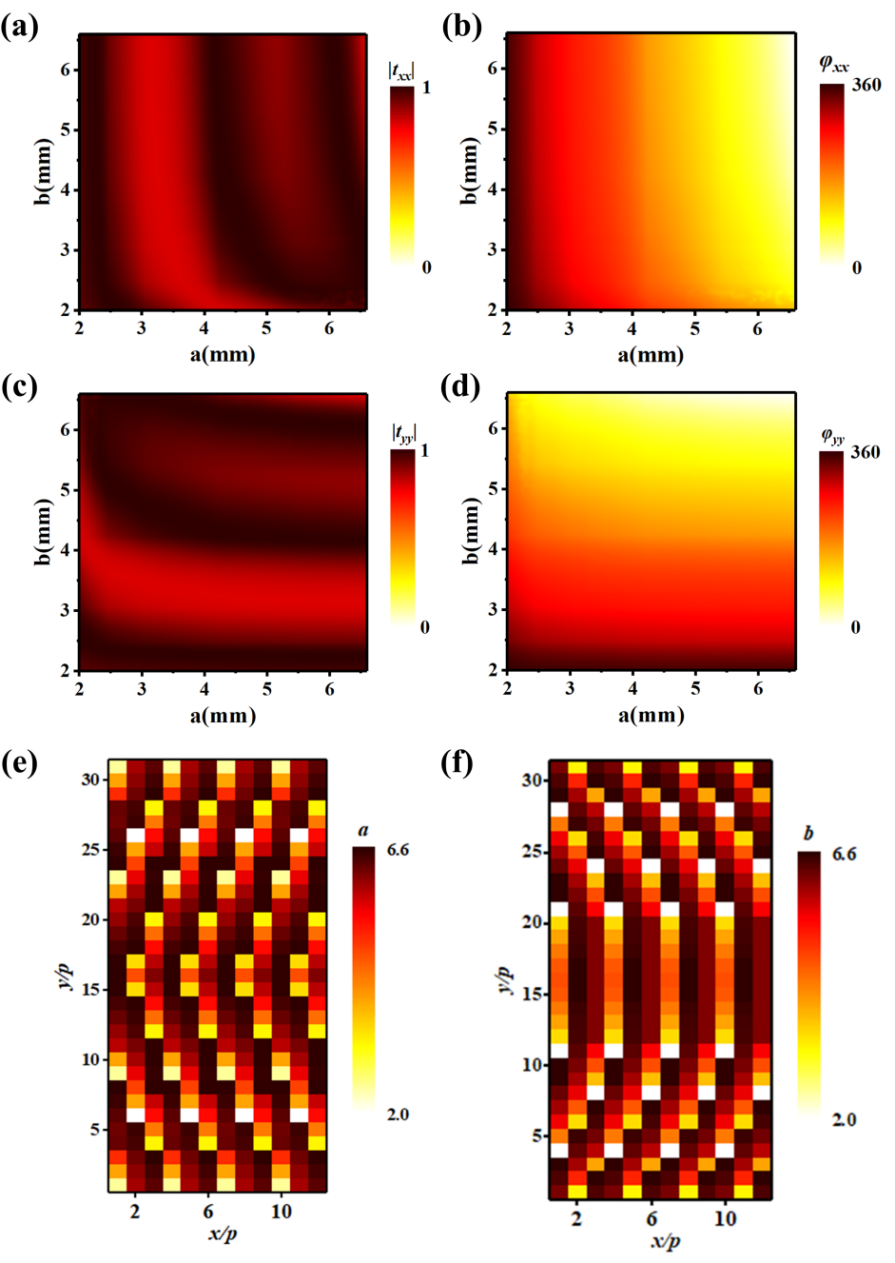


**Figure S2 Maps of transmission amplitude/phase of the metasurface and detailed structural parameters of the TPGM.** Transmission amplitude (a) $|t_{xx}|$, (c) $|t_{yy}|$ and transmission phase (b) $\varphi_{xx}$, (d) $\varphi_{yy}$ as functions of *a* and *b* at 10.5 GHz. Distributions of the parameters (e) *a* and (f) *b* for the designed TPGM.

1. **Design and details of the composite SSPP eigenmode plate**

Traditionally, the design of an anisotropic eigen-unit is aimed at supporting both TM- and TE-mode SSPPs only propagating along the phase gradient direction (i.e., just planar wavefront). However, the dispersion of the anisotropic eigen-unit is sensitive to the travel direction of SSPP (i.e., the functional wavefront of SSPP), which leads to momentum mismatch of functional SSPP, as depicted in Fig. S3(a). Here, with the wave-vector being 1.12*k*_0_ to match the phase gradient of the TPGM, we calculated both the TM- and TE-mode eigenfrequencies of the anisotropic eigen-unit with different rotate angles (i.e., different travel directions of SSPP). It can be seen that the eigenfrequencies varied with the rotate angles resulting in momentum mismatch. So, we design two kinds of SSPP eigenmode plates made of isotropic eigen-units at both sides to match the TM- and TE-mode SSPPs, respectively. It can improve the whole efficiency while suppressing the crosstalk between different polarities. Figure S3(b) shows the eigenfrequencies of the two isotropic eigen-units with different rotate angles, which are insensitive to the angles. As for the middle area of the SSPP eigenmode plate, the anisotropic eigen-unit is necessary to support both the TM- and TE-mode SSPPs. Moreover, to verify the above analysis, Figs. S3(c) and S3(d) depict the FDTD simulated TE-mode SSPP focusing beam and TM-mode SSPP Bessel beam with the anisotropic SSPP eigenmode plate, respectively. Figure S3(e) shows the simulation model of the bifunctional meta-coupler. Compared with the results of our employed composite SSPP eigenmode plate in the manuscript, the effects of the SSPP Bessel beam and focusing beam in Figs. S3(c) and S3(d) are deterioration due to the momentum mismatch.


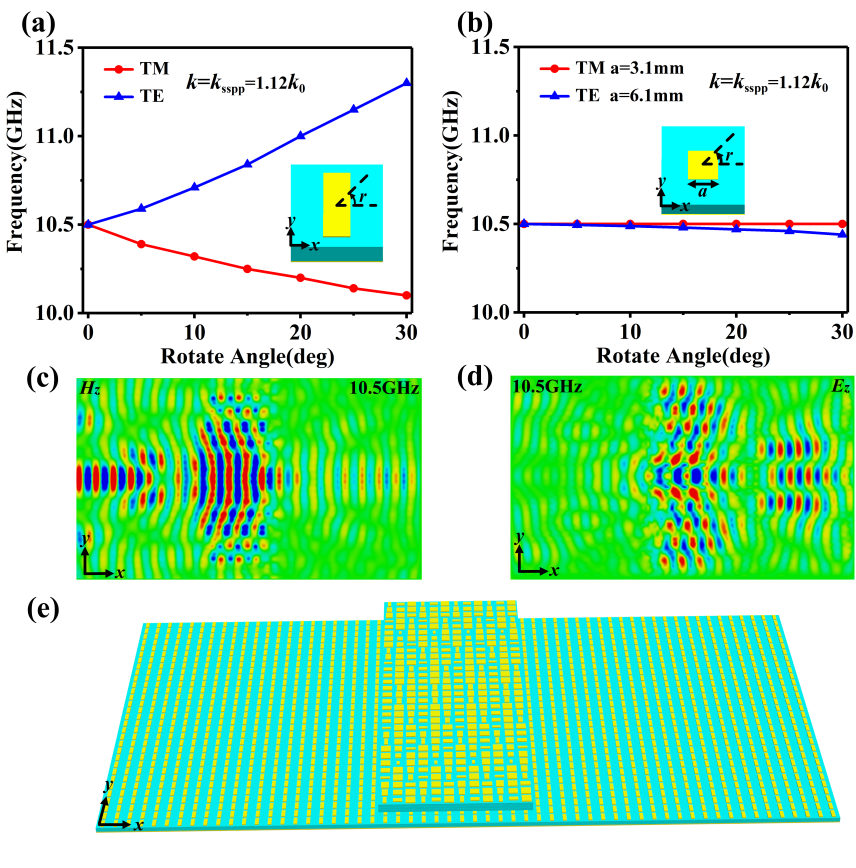


**Figure S3** (a)-(b) The simulated TM- and TE-mode eigenfrequencies of the (a) anisotropic eigen-unit and (b) two isotropic eigen-units versus the rotate angle, when wave-vector being *k*_sspp_. (c)-(d) The FDTD simulated (c) TE-mode SSPP focusing beam and (d) TM-mode SSPP Bessel beam with the anisotropic SSPP eigenmode plate. (e) Schematic diagram of the bifunctional meta-coupler with an anisotropic SSPP eigenmode plate.

1. **Simulation results and cross talk for the bifunctional SSPP meta-coupler**

Here, we employ FDTD simulations to verify the principle and design for our bifunctional SSPP meta-coupler integrating the Bessel beam and focusing effect. Figures S4(b) and S4(d) demonstrate the simulated $Re(E_{z})$ field patterns on the *x-y* plane (with *z* = 6 mm) and on the *x-z* plane (with *y* = 0) in the meta-coupler respectively, when it is shined by an *x*-polarized wave. The simulated results clearly illustrate that the *x*-polarized wave is first converted to SSPP corresponding with the theoretically designed wave-vector $k_{\mathrm{sspp}}$. And then, the excited SSPP travels to the right and interferes with each other to form the SSPP Bessel beam.

Similarly, Figs. S4(a) and S4(c) depict the operating mechanism of the meta-coupler converting the incident *y*-polarized wave into the leftward propagating TE-mode focusing SSPP with an obvious focal point. The $Re(H_{z})$ field patterns are plotted on the same two planes with that $Re(E_{z})$ field patterns except that they are on the left side.

Moreover, we have investigated the crosstalk of the SSPP Bessel and focusing beam, respectively. Figures S4(e) and S4(g) show the simulated $Re(E_{z})$ field patterns of the TE SSPP focusing beam on the *x-y* plane (with *z* = 6 mm) and on the *x-z* plane (with *y* = 0) in the meta-coupler respectively. And Figs. S4(f) and S4(h) show the simulated $Re(H_{z})$ field patterns of the TM SSPP Bessel beam on the same two planes. As we can see, the crosstalk fields of the SSPP Bessel and focusing beam are very weak compared with the results of the main polarization, which is mainly due to the fact that our composite SSPP eigenmode plate has isotropic eigen-units on both sides for TM SSPP Bessel and TE SSPP focusing beam, respectively. Summarily, both the $Re(E_{z})$ and $Re(H_{z})$ field patterns exhibit excellent performances without other cluttered field distributions and other crosstalk between different polarities, which agree well with our principle and theoretical designs and indicate high efficiencies of the meta-coupler.


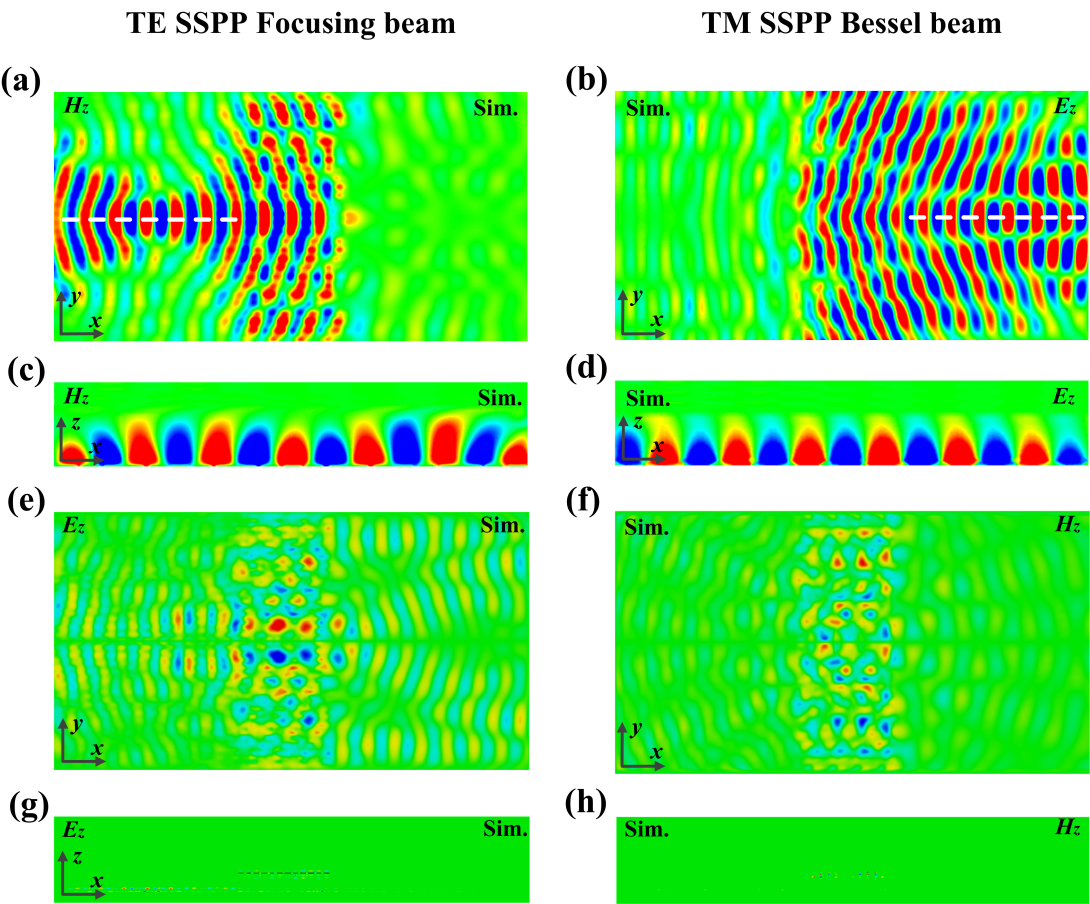


**Figure S4 FDTD near-field simulation results of the bifunctional SSPP meta-coupler.** Simulated $Re(H_{z})$ field patterns of the TE SSPP focusing beam on the (a) *x*-*y* plane (with *z* = 6 mm) and (c) *x*-*z* plane (with *y* = 0 mm). Simulated $Re(E_{z})$ field patterns of the TE SSPP focusing beam on the (e) *x*-*y* plane (with *z* = 6 mm) and (g) *x*-*z* plane (with *y* = 0 mm). Simulated $Re(E_{z})$ field patterns of the TM SSPP Bessel beam on the (b) *x*-*y* plane (with *z* = 6 mm) and (d) *x*-*z* plane (with *y* = 0 mm). Simulated $Re(H_{z})$ field patterns of the TM SSPP Bessel beam on the (f) *x*-*y* plane (with *z* = 6 mm) and (h) *x*-*z* plane (with *y* = 0 mm). White dashed lines denote the positions of the *x*-*z* plane.

1. **The working efficiency of the bifunctional SSPP meta-coupler**

To quantitatively characterize the performance of our bifunctional SSPP meta-coupler, Figs. S5(c) and S5(d) depict the working efficiency of the TE SSPP focusing beam and TM SSPP Bessel beam, respectively. The working efficiency is defined as the ratio between the power carried by the SSPP functional beam (SSPP Bessel or focusing beam) and that carried by the total incident power. The total incident power is calculated by integrating the power illuminated on the TPGM, and the power of the SSPP functional beam is calculated by integrating the power passing through the SSPP wavefront plane (see Figs. S5(a) and S5(b)). In the simulations, the *x*- and *y*-polarized waves with *E*-field amplitudes 1 V/m are incident on the TPGM, and the power flow monitor is employed to obtain the corresponding power flow distributions of the SSPP functional beam and the incident beam at different frequencies. Then the power of the SSPP functional beam and the incident waves can be calculated by summing the power flow on the TPGM and the SSPP wavefront plane with a proper step size. In order to ensure that the calculated power is convergent and thus correct, the step size should be as small as possible, which can be verified by the consistency of the calculated efficiency values under two different step sizes. Based on the above method, a convergent step size of 0.5 mm is employed to calculate the corresponding power. The power of the incident beam is calculated by summing the power flow on the white rectangular area of TPGM, when there are only incident waves on the TPGM, which can be achieved by setting the whole model as vacuum in the simulations. As for the power of the SSPP focusing beam and Bessel beam, power flows on the SSPP focusing plane (see Fig. S5(a)) and SSPP Bessel plane (see Fig. S5(b)) are summed, respectively. Here, the simulated working efficiency of the TE SSPP focusing beam and TM SSPP Bessel beam at 10.5 GHz is 57% and 60%, respectively. The missing power is partly taken by the scattered energy to the far-field (i.e., total reflection *R*), partly taken by the structure absorption, and the cluttered SSPP. As for the measured efficiency, it is difficult to quantitatively describe it since the measurement of the power flow is a great challenge. Despite this, due to the measured near-field and far-field results agreeing well with the simulated ones, we can qualitatively surmise the good efficiency of measurement.

Moreover, to further quantitatively characterize the crosstalk between different polarities of both TE SSPP focusing beam and TM SSPP Bessel beam, Figs. S5(c) and S5(d) also depict the crosstalk efficiency of the TE SSPP focusing beam and TM SSPP Bessel beam, respectively. The crosstalk efficiency is defined as the ratio between the power carried by the SSPP functional crosstalk beam and that carried by the total incident power. Here, the simulated crosstalk efficiency of the TE SSPP focus beam and TM SSPP Bessel beam at 10.5 GHz is 4% and 2%, respectively. It can be seen that the crosstalk of the SSPP Bessel and focusing beam is very little so that there is little energy will be lost in another channel since our composite SSPP eigenmode plate is made of two isotropic eigen-units to match the TM SSPP Bessel and TE SSPP focusing beam, respectively.


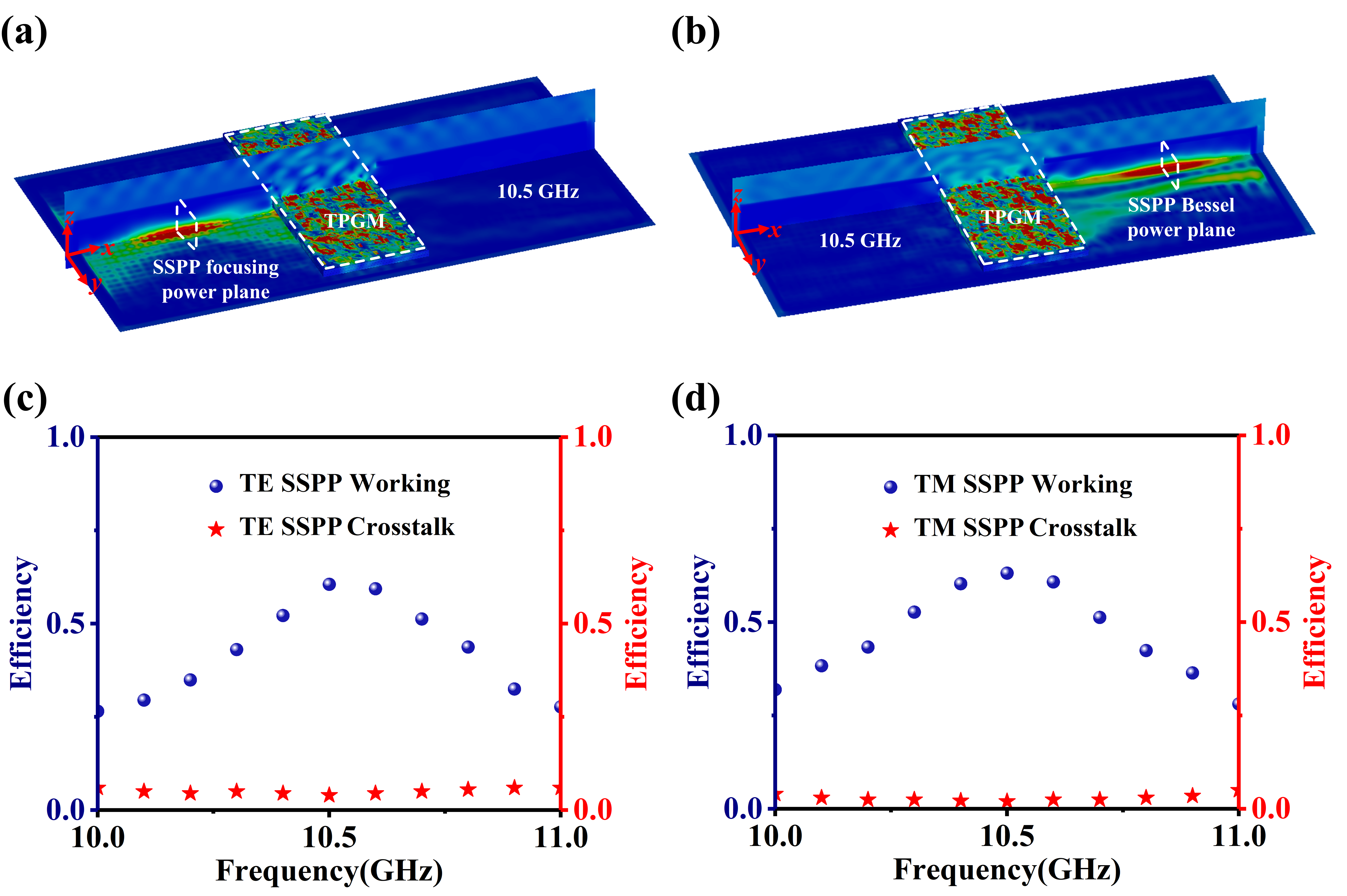


**Figure S5** (a-b) Simulated power flow distributions at 10.5 GHz on the *x*-*y* plane (with *z* = 6 mm) and *x*-*z* plane (with *y* = 0 mm) of the (a) TE SSPP focusing beam and (b) TM SSPP Bessel beam. The two white rectangular areas in *y*-*z* plane of (a) and (b) are SSPP focusing power plane and SSPP Bessel power plane, respectively. The two white rectangular areas in *x*-*y* plane of (a) and (b) are both the TPGM plane, which is for the calculation of the total incident power when there are only incident waves on the TPGM. (c-d) The working efficiency and crosstalk efficiency of the (c) TE SSPP focusing beam and (d) TM SSPP Bessel beam.

1. **Simulated efficiency of the meta-coupler versus the distance *d* between the metasurface and the SSPP eigenmode plate**

In this paper, to generate both TM- and TE-mode SSPP, we designed a transmissive SSPP coupler in which the SSPP eigenmode plate is placed at a distance of *d* under the TPGM. Figure S6 depicts the FDTD simulated efficiency of the meta-coupler versus the distance *d* between the metasurface and the SSPP eigenmode plate at 10.5 GHz. It can be seen that the distance *d* has an influence on the SSPP efficiency, and the highest efficiency can be obtained when *d* is fixed at a particular value *d*_c_=12 mm.


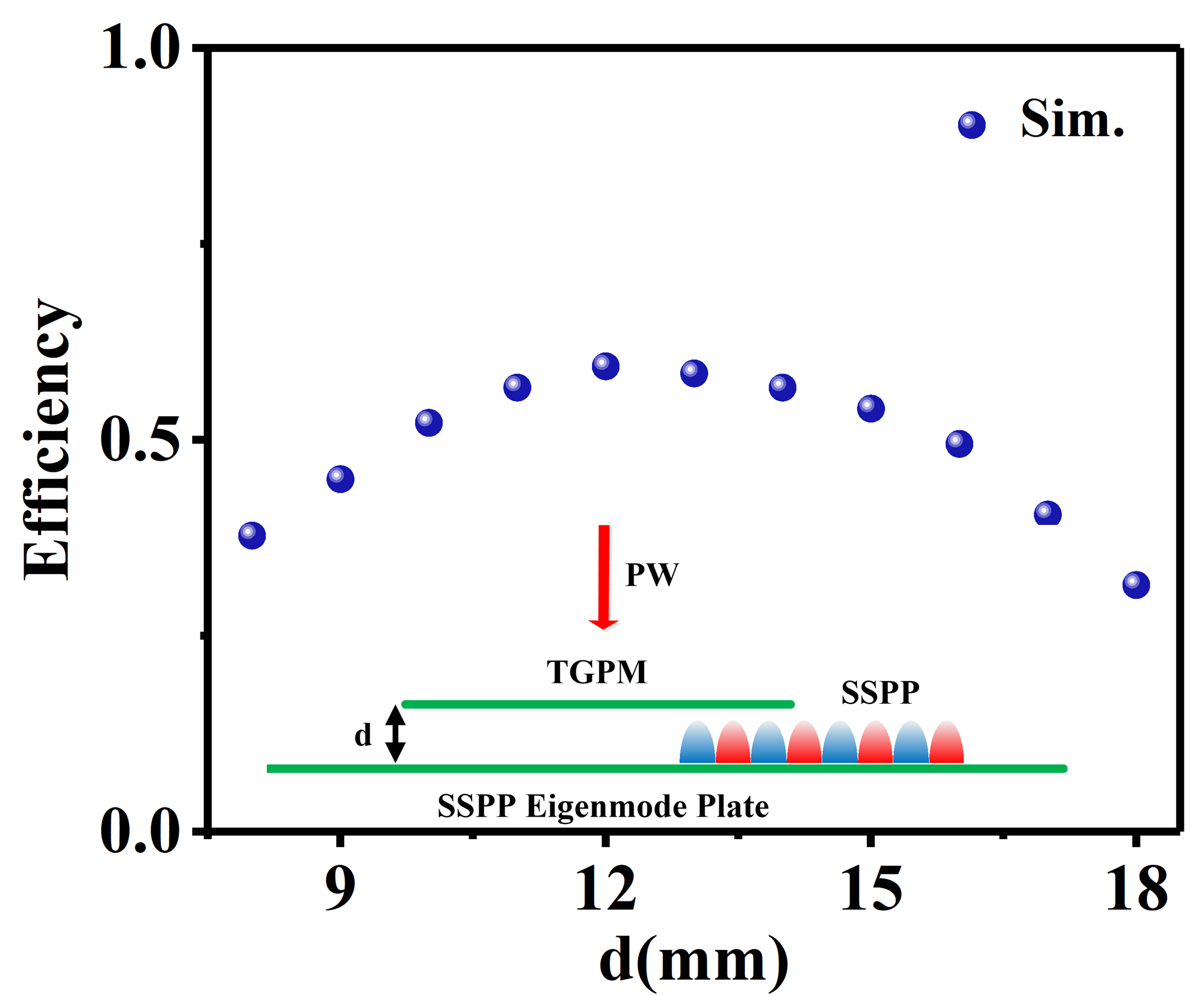


**Figure S6** Simulated efficiency of the meta-coupler versus the distance *d* between the metasurface and the SSPP eigenmode plate at 10.5 GHz.
